# Neuroprotective Effect of Low-Intensity Transcranial Ultrasound Stimulation in Moderate Traumatic Brain Injury Rats

**DOI:** 10.3389/fnins.2020.00172

**Published:** 2020-03-10

**Authors:** Tao Zheng, Juan Du, Yi Yuan, Shuo Wu, Yinglan Jin, Zhanqiu Wang, Defeng Liu, Qinglei Shi, Xiaohan Wang, Lanxiang Liu

**Affiliations:** ^1^Department of Magnetic Resonance Imaging, Qinhuangdao Municipal No. 1 Hospital, Qinhuangdao, China; ^2^Institute of Electrical Engineering, Yanshan University, Qinhuangdao, China; ^3^Peking University Health Science Center, Beijing, China; ^4^Siemens Ltd., Beijing, China

**Keywords:** LITUS, traumatic brain injury, ultrasound, therapy, neuroprotective

## Abstract

Traumatic brain injury (TBI) is a kind of severe brain injury characterized with a high incidence rate and a high disability rate. Low-intensity transcranial ultrasound stimulation (LITUS) is a promising neuroprotective method for improving the functional prognosis of TBI. The fractional anisotropy (FA) value and mean diffusivity (MD) value can be sensitive to abnormal brain structure and function and can thus be used to evaluate the effect of LITUS on TBI. Our purpose was to evaluate the therapeutic effect of LITUS in a moderate TBI rat model with FA and MD values. For our method, we used 45 male Sprague Dawley rats (15 sham normal, 15 TBI, and 15 LITUS treatment rats). We used single-shot spin echo echo-planar imaging sequences at 3.0T to obtain the DTI parameters. Parameters of FA and MD on the treated side of the injury cortex were measured to evaluate the therapeutic effect of LITUS in a TBI rat model. For FA and MD values, groups were compared by using a two-way analysis of variance for repeated measures, and this was followed by Tukey’s *post hoc* test. Differences were considered significant at *P* < 0.05. The results were that the FA value in the LITUS treatment group at 1 day after TBI was significantly higher than that in the control group (adjusted *P* = 0.0422) and significantly lower than that in the TBI group at 14, 21, and 35 days after TBI (adjusted *P* = 0.0015, 0.0064, and 0.0173, respectively). At the end of the scan time point, the differences between the two groups were not significant (adjusted *P* = 0.3242). The MD values in the LITUS treatment group were significantly higher in the early stage than that in the TBI group (adjusted *P* = 0.0167) and significantly lower at the following time points than in the TBI group. In conclusion, daily treatment with LITUS for 10 min effectively improved the brain damage in the Controlled Cortical Impact (CCI)-caused TBI model. FA and MD values can serve as evaluation indicators for the neuro-protective effect of LITUS.

## Introduction

Traumatic brain injury (TBI) is a form of brain injury caused by trauma, and it has a high incidence and a high rate of disability. Recent epidemiological studies have shown that more than 40 percent of people experience varying degrees of TBI, from mild to severe forms, during their lifetime (Zetterberg et al., 2013; Williams et al., 2018). TBI can lead to several forms of neurological dysfunction, including motor impairment, personality changes, vascular dementia (VaD), and Alzheimer’s disease (AD). There is also a positive correlation between the degree of injury and the severity of complications. Therefore, it is important to research and implement efficient interventions for TBI in order to reduce the abovementioned complications (Scott et al., 2009; Hayes et al., 2017; Kowalski et al., 2017).

In recent decades, the development of low-intensity pulsed transcranial ultrasound (LITUS) has made great strides in the field of neurotherapy. LITUS uses low-energy sound waves to penetrate the skull and skin in a non-surgical condition, focusing on a precise area in the brain and thereby regulating neural activity. Compared to other physical therapy technologies, ultrasound therapy has several advantages including accurate focusing and ease of use. It, therefore, has the potential to be utilized as a safer clinical application of continuous therapy. Studies have demonstrated LITUS’s regulatory effects on epilepsy, depression, anxiety, and other neurological disorders (Stuart et al., 2013; Panczykowski et al., 2014). Our previous study found that LITUS has a regulatory effect on the diffusion of brain water molecules, and this leads to a change in the apparent diffusion coefficient (ADC) value of brain tissue in the LITUS stimulation region (Yuan et al., 2017). Recent studies have shown that LITUS brain stimulation can awaken patients with severe post-TBI consciousness disorders, and patients can recover successfully under ultrasound stimulation (Monti et al., 2016). Therefore, LITUS is a promising type of physical therapy for brain trauma.

Current commonly used clinical indicators for the diagnosis of TBI and evaluation of its prognosis include the Glasgow scale evaluation and imaging examinations (mainly CT). Clinical practices, however, have demonstrated that these evaluation indicators are not always ideal for the diagnostic and prognostic prediction of TBI patients. The severity of TBI is assessed using the Glasgow coma scale. This method can rapidly determine the degree of consciousness disturbance in patients with TBI and predict the prognosis of patients by assessing the opening of eyes, reaction time, and speech. However, this scaling method is subject to patient bias and does not reflect the functional status of the brain stem. It is also difficult to evaluate children, the elderly, and patients with endotracheal intubation (Joseph et al., 2015). CT is the most commonly used imaging method to evaluate post-TBI. However, TBI does not only cause pathological changes at the macro level, such as local brain tissue contusions and bleeding, but also cause pathological changes at a micro level, such as axonal fractures and microhemorrhages (Immonen et al., 2009; Wei et al., 2012). CT fails to detect these microlesions and often underestimates the degree of injury in patients (Le and Gean, 2010). With the development of imaging technology, many researchers have found MRI to accurately evaluate brain tissue damage after TBI, and its application value in TBI has garnered a great deal of attention. Recently, researchers have found that LITUS greatly improves the degree of cerebral edema in TBI rats (Su et al., 2015, 2017a,b). However, their study only used the T2WI imaging method commonly used in animal studies and has limited sensitivity to the injury of brain fiber bundles commonly observed in TBI. As an emerging fiber imaging technology, magnetic resonance diffusion tensor imaging (DTI) enables researchers to quantify the signal data of diffusion anisotropy by measuring such parameters as mean diffusivity (MD) and fractional anisotropy (FA), in order to show the damage to white matter fiber bundles caused by damage (Cascio et al., 2007). Focal or diffuse axonal discontinuity in the brain, caused by trauma, is the key reason behind brain neuropathy and cognitive changes (Xiong et al., 2014). Therefore, DTI examination is of great value in the diagnosis and prognosis of TBI. This study treated TBI model rats with LITUS and evaluated the efficacy using FA and MD values.

## Materials and Methods

### Rats

Forty-five male Sprague Dawley rats with an average weight of 250 g and an average age of 2 months were randomly divided into three groups (see below). Animals were housed at 20–22°C in 60% air humidity and with *ad libitum* access to food and water. All rats were given sodium pentobarbital (3%, 5 mg/100 g, IP, Cat.No.P8410, Beijing Solarbio Tech Co., Ltd.) prior to surgery. The experimental conditions were in accordance with the international ethical statutes and law for the protection of animals and were approved by the Medical Ethics Committee of Qinhuangdao First Hospital.

Forty-five rats were randomly divided into 3 groups. Rats in the TBI group (*n* = 15) received a Controlled Cortical Impact (CCI) operation at the beginning of the study. The model of moderate TBI in rats was established by the Feeney free fall device (eCCI Model 6.3; Custom Design, Richmond, VA, United States). The impact device had several parameters: the impact hammer weight was 21 g, the impact pipe length was 26 cm, the impact bar diameter was 4.6 mm, and the end extended 4 mm beyond the lower edge of the catheter. Freefall blows using these parameters results in moderate traumatic brain injury (Pan et al., 2019). Humane care was given to the rats according to the 3R principles of experimental animals, and the rats were weighed according to the aseptic operation procedures. Rats were anesthetized using an intraperitoneal injection with 10% chloral hydrate 4 ml/Kg and kept warm by light. The rats were fixed to the operating table, the hair on the scalp was cut off, and the skin was sterilized with 75% alcohol. An opening was cit 1.5–2 cm long along the parietal bone, and skin was removed to expose the parietal bone. A small hole was created with a diameter of 5 mm at the skull 2.5 cm to the right of the midline and 1.5 cm behind the anterior fontanelle (keep the dura intact). The dura mater was then struck with the tip of the Feeney free fall device. The hammer lifted immediately after the blow. If bleeding occurred during the operation, a cotton swab was used immediately to provide pressure to stop bleeding. The surgical field was cleaned, and the incision was sutured immediately. The rats were first transferred to a thermal pad and then returned to the cage after physical activity. Rats in the LITUS treatment group (*n* = 15) received the same operation. After the CCI operation, the rats were then treated with LITUS stimulation once a day for 42 days. The details of the LITUS methods are described below in section “LITUS Protocol.” Rats in the Sham Control group (*n* = 15) received a scalp incision and cranial drilling without any CCI operation or LITUS stimulation.

### LITUS Protocol

**FIGURE 1:**
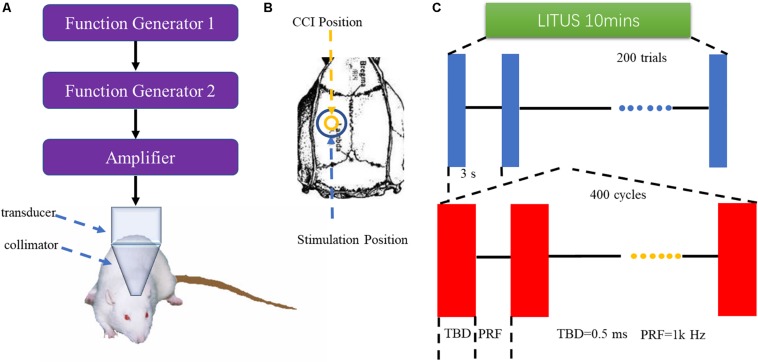
**(A)** LITUS system, two connected function generators were used to generate pulsed signals. The pulsed signal from the second generator was amplified by a linear power amplifier and transmitted to an unfocused ultrasound transducer. The ultrasound transducer was connected to the rat skin by a conical collimator with a diameter of 10 mm that was filled with ultrasound coupling gel. **(B)** Stimulation position and CCI position. **(C)** Time schedule of ultrasound stimulation and the parameters of the ultrasound.

The pulsed ultrasound setup was similar to that used in our previous study (Liu et al., 2019). As shown in Figure 1, two function generators (AFG3022C; Tektronix, United States) were used to generate ultrasonic pulse signals, which were then amplified by power amplifiers (E&I240 L; ENI Inc., United States) and transmitted to ultrasonic transducers (V301-SU; Olympus, Japan). The LITUS then collimated to the rat brain. The area of the unfocused ultrasound transducer was 3.5 cm^2^. The total stimulation duration was 10 min with 200 trials. The ultrasound fundamental frequency (FF) and pulse repetition frequency (PRF) were 500 and 1 kHz, respectively. The ultrasound stimulation duration (SD) and tone-burst duration (TBD) parameters were 400 and 0.5 ms, respectively. The ultrasound pressure was measured by a calibrated needle-type hydrophone (HNR500, Onda, Sunnyvale, CA, United States), and the spatial peak and pulse-average intensity (Isppa) was 2.6 W/cm2.

### MR Image Acquisition

The rats were scanned using a 3.0-Tesla Siemens TIM Verio Scanner (Siemens Medical Solutions, Erlangen, Germany) at days 1, 7, 14, 21, 28, 35, and 42 after TBI. Special animal MRI coils (4 channel high resolution, diameter 50 mm, P/N 10-F04885, Shenzhen RF Tech Co., Ltd.) were used in this study. Each rat head was held by a specific method: a string was tied to the incisors of the rats, and the other end of the string was tied to a water phantom to ensure that the head could not move during the scanning. All rats were given sodium pentobarbital (3%, 5 mg/100 g, IP, Cat.No.P8410, Beijing Solarbio Tech Co., Ltd.) prior to the MR scan. The image quality was analyzed after the MR scanning; if the rat’s head moved during the scanning, the scan was repeated 2 h later with an additional suitable amount of anesthetics. Below are detailed parameters of the MR sequences.

#### Axial T2WI

For the MR parameters, slices were aligned parallel to the anterior/posterior line with various settings: TR = 4000 ms, TE = 113 ms, flip angle = 150°, average = 6, FOV = 80 × 80 mm, voxel size = 0.3 × 0.3 × 2.0 mm, data matrix = 192 × 192, slice thickness = 2.0 mm, and the number of slices = 10 (total scanning time = 3 min 18 s).

#### Resolve-DTI

The RESOLVE technique was performed to acquire DTI images. For the MR parameters, slices were aligned parallel to the anterior/posterior line with the following settings: TR = 2000 ms, TE = 100 ms, flip angle = 150°, FOV = 124 × 124 mm, voxel size = 2.3 × 2.3 × 2.0 mm, data matrix = 64 × 64, slice thickness = 2 mm, number of slices = 10, diffusion directions = 20, *b*-values = 0, and 1,000 s/mm^2^, respectively (total scanning time = 17 min 12 s). A multi-segmented k-space filling technique was used in this sequence, which reduced the echo spacing of two adjacent echoes. At the same time, an echo navigation technique and a motion threshold monitoring technique were used to improve the image quality.

#### MR Image Post-processing and Analysis

**FIGURE 2:**
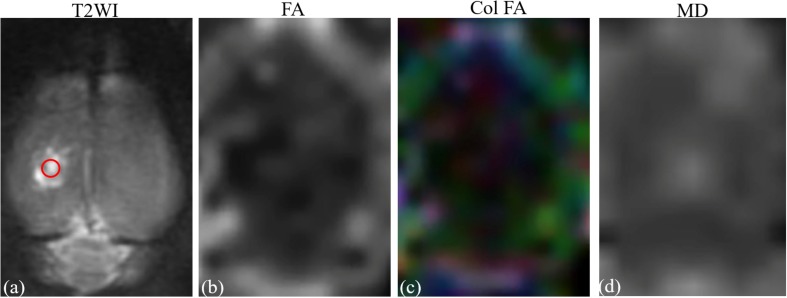
**(a)** T2-weighted image. **(b)** Fractional anisotropy map. **(c)** Color fractional anisotropy map. **(d)** Mean diffusion map. ROI of injury area (red circle in A) was chosen on axial T2-weighted image with which the fractional anisotropy map and T2-weighted image co-registered.

Imaging analysis was carried out using a prototype software on a workstation (Siemens Verio 3.0T MR Leonardo 3682). All rats were given sodium pentobarbital (3%, 5 mg/100 g, IP, Cat.No.P8410, Beijing Solarbio Tech Co., Ltd.) prior to the MR scan. The FA and MD parameter maps of rat brains were co-registered with T2WI rat brain images for the exact identification of the damaged cortex and contralateral mirrored area. Two independent radiologists, with 10 years of experience in neural MRI, were blinded to the animal grouping and placed circular regions of interest (ROIs) measuring 0.30–0.60 cm^2^ in the center of the damaged cortex on T2WI rat brain images for the TBI (Figure 2). The FA and MD image maps were strictly co-registered with the T2WI images to accurately measure the FA and MD values of the damaged area. For the LITUS treatment group, the FA and MD values were measured using the same method for the exact identification of the cortex. For the control group, the same position of cortex was selected. In addition to the focal lesion, FA and MD values at the contralateral mirrored cortex were also measured using the same method to verify the stability of FA and MD parameters. The mean values of repeated measurements of the two sides were used in the final analysis. Edema volumes of TBI and LITUS treatment groups were assessed from T2-weighted images by summing up the edema area measured from six slices and multiplying by the slice thickness.

### Immunohistochemical Staining

After the last MR scan (42 days after TBI modeling), the rats were sacrificed under ether anesthesia and the brains were collected for HE and Nissl staining.

#### HE Staining

After the sections were dewaxed by xylene, the sections were dehydrated with gradient alcohol and stained with hematoxylin dye for 1–2 min. The nuclei were then stained with hematoxylin for 5 min, and then washed again. After staining with an eosin solution for 10 min, the samples were dehydrated with gradient alcohol and sealed with neutral gum. The samples were then subjected to microscope observation, photographed, and archived. Each stained section was examined by a research team member with over 10 years’ experience using light microscopy.

#### Nissl Staining

Brains from the three groups were removed intact and fixed in 4% paraformaldehyde overnight at 4°C and then placed in the cryoprotection solution of 30% sucrose for 2–3 days at 4°C until they were no longer buoyant. Coronal sections were made with reference to bregma: they started from 1 to 2 mm behind the anterior fontanelle and cut on a cryostat (−20°C) at a thickness of 8 μm and were mounted on slides. Neuronal density was quantified using Nissl staining with Cresyl violet. Frozen slides were air-dried for 1 h, stained in Cresyl violet solution (0.1% Cresyl violet) for 5 min, rinsed in distilled water, incubated in 95% ethyl alcohol for 15 min, dehydrated in 100% alcohol for 5 min twice, twice cleared in xylene for 5 min, and then mounted by resinous medium for the quantification. Each stained section was examined by a research team member with over 10 years of experience using light microscopy.

#### Immunohistochemical Staining Image Analysis

Stainings were scored according to the procedure described by Axiotis et al. (1991), with minor modifications. Twenty high-power fields were randomly chosen for each cortex of the rats in the three groups. The percentage of Nissl cells and intensity of immunostaining were assessed by two independent immunologists with 10 years of experience. TH immunostaining was semi-quantitatively expressed as the percentage of TH+cells: 0, 0–10%; 1+, 11–25%; 2+, 26–50%; 3+, 51–75%; 4+, and 76–100%. It was also expressed as intensity of immunostaining: 1 +, weak staining; 2 +, moderate staining; 3 +, and strong staining. The results of these two scoring methods were added to create grading scores: 0–2 representing weak expression, 3–5 representing moderate positive expression, and 6–7 representing the strongest positive expression. The mean values of the 20 high-power fields were also calculated, and the results were rounded to the nearest whole number.

### Behavioral Tests

The neuroscore showed that Mice were scored from 4 (normal) to 0 (severely impaired) for (1) forelimb function while walking on the grid and flexion response during suspension by the tail; (2) hind limb function while walking on the grid and extension during suspension by the tail; and (3) resistance to lateral right and left push. The best score possible was 12.

### Statistical Analysis

Statistical analyses were performed using standard software packages (GraphPad Prism version 7.00, La Jolla, CA, United States). Data are presented as mean ± SD. The normality of the data was tested and confirmed by Shapiro–Wilk tests. The intraclass correlation coefficients of the repeated two-time measurements were calculated. For the neuroscore, the FA and MD values, groups were compared using a two-way analysis of variance for repeated measures followed by the Tukey’s *post hoc* test. For histological analysis, groups were compared using the unpaired *t*-test. Differences were considered significant at *P* < 0.05.

## Results

All the intraclass correlation coefficients of measured results from the two radiologists were greater than 0.8, which indicates a good correlation. The average values of repeated measurements of FA and MD values were taken as the final values of FA and MD.

### FA and MD Values Dynamically Evaluate the Recovery of Brain Injury Rats

#### FA of Injury Cortex in TBI, LITUS Treatment, and Sham Control Groups

**FIGURE 3:**
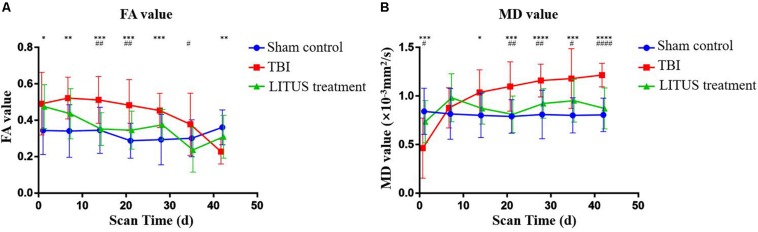
**(A)** Trends of FA values in the injury cortex. **(B)** Trends of MD values in injury cortex. Two-way ANOVA for repeated measurements, followed by Tukey’s *post hoc* test. *P < 0.05, **P < 0.01, ***P < 0.001, and ****P < 0.0001, sham control vs. TBI. #*P* < 0.05, ##*P* < 0.01, ###*P* < 0.001, and ####*P* < 0.0001, TBI vs. LITUS treatment. Mean ± SD, *n* = 15.

For the Sham Control group, the FA value did not change at each time point, and the curve remained constant. For the TBI group, compared to the sham control group, the overall FA value showed an increasing trend in the early stage and a decreasing one in the later stage. FA value at 1 day after injury was significantly higher than that in the sham control group (adjusted *P* = 0.0202), which slowly decreased over time and was significantly lower than the normal rats (adjusted *P* = 0.0014) at the end of the scan (adjusted *P* = 0.0202). In the LITUS treatment group, the FA value at 1 day after TBI was significantly higher than that in the control group (adjusted *P* = 0.0422), and the FA value at days 14, 21, and 35 after TBI were significantly lower than that in the TBI group (adjusted *P* = 0.0015, 0.0064, and 0.0173, respectively). At the end of the scan time point (day 42), the differences between the two groups were not significant (adjusted *P* = 0.3242) (Figure 3A and Supplementary Table 1).

#### MD of Injury Cortex in TBI, LITUS Treatment, and Sham Control Groups

Similar to the FA value curve for the sham control group, the MD value did not change at each time point, and the curve remained constant. Compared to the sham control group, MD value in the TBI group showed a significant decrease during the early stage and a significant increase in the late stage. MD value was significantly decreased 1 day after injury, which was lower than that in the Sham Control group and the LITUS treatment group (adjusted *P* = 0.0167 and 0.0007, respectively). A slight increase in MD value was observed at day 7 and was significantly higher than that in the normal group at all subsequent time points. In the LITUS group, the curves were similar to those in the sham control group, which were significantly higher in the earlier (day 1) stage than in the TBI group (adjusted *P* = 0.0167), and significantly lower at the subsequent time points than in the TBI group (Figure 3B and Supplementary Table 2).

#### FA and MD of Contralateral Mirror Cortex in TBI, LITUS Treatment, and Sham Control Groups

By measuring the FA and MD values of cortex of the same brain region on the contralateral mirror cortex, we found that there was no significant difference in FA and MD between TBI, LITUS, and Sham control groups (all adjusted *P* > 0.05). At the same time, the values of FA and MD in the three groups were stable in the baseline state, without any increase or decrease (Supplementary Tables 3, 4). The results laterally confirmed the reliability of FA and MD measurements on the lesion side.

#### FA and MD Values at the Focal Lesion and Contralateral Mirror Cortex in TBI, LITUS Treatment, and Sham Control Groups

For the TBI group, FA values of the focal lesion were higher than that of the contralateral mirror cortex at day 1, 7, 14 and 21 (adjusted *P* = 0.0014, 0.0001, 0.0001, and 0.0015, respectively). MD values of the focal lesion were higher than contralateral mirror cortexat day 1 (adjusted *P* = 0.0006) and lower than that of the contralateral side at days 14, 28, 35, and 42 (adjusted *P* = 0.0431, 0.0456, 0.0270, and 0.0165, respectively). For the LITUS treatment group, there was a significant difference in FA value for day 1 (adjusted *P* = 0.0174), but no difference at other time points. There was no significant difference in MD value at each time point. For the Sham control group, there was no significant difference for FA and MD values between the focal lesion and the contralateral mirror cortex at each time point (Supplementary Tables 5, 6).

#### Brain Edema Volume of Injury Cortex in TBI and LITUS Treatment Groups on T2WI

**FIGURE 4:**
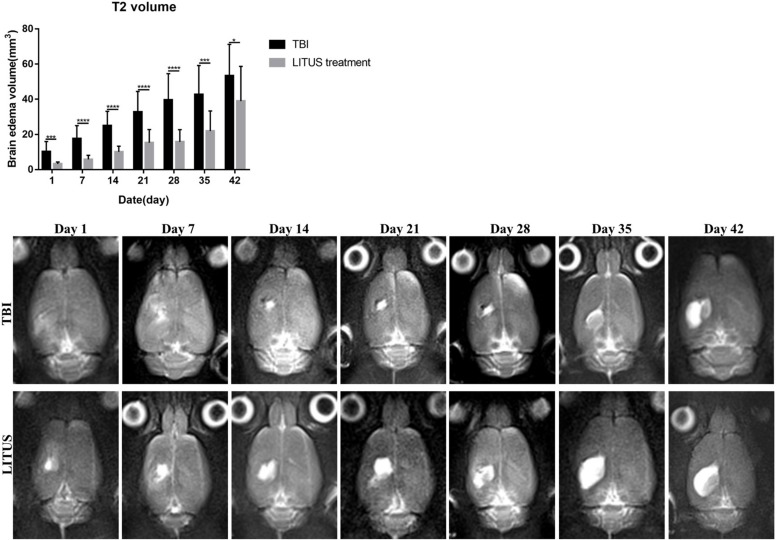
Effects of LIPUS treatment on brain edema in TBI rats. Representative T2-weighted MRI images at days 1, 7, 14, 21, 28, 35, and 42 post-TBI. The damaged area is defined as a hyperintense region over the right cortex, indicating edema formation (*P < 0.05, ***P < 0.001, ****P < 0.0001, *n* = 15).

The volume of edema in the TBI and LITUS groups was 10.2 ± 5.7 and 17.6 ± 7.4, respectively, 1 day after CCI (adjusted *P* = 0.0003). Then the volume of brain edema increased with time. At 42 days, it was 53.3 ± 17.9 and 38.9 ± 19.8, respectively (adjusted *P* = 0.0452). During the whole observation period, the edema volume of LITUS group was always smaller than that of LITUS group. Neurological function score dynamically display the recovery of motor function in rats during the observation period (Figure 4).

### Neurological Function Score Dynamically Display the Recovery of Motor Function in Rats During the Observation Period

**FIGURE 5:**
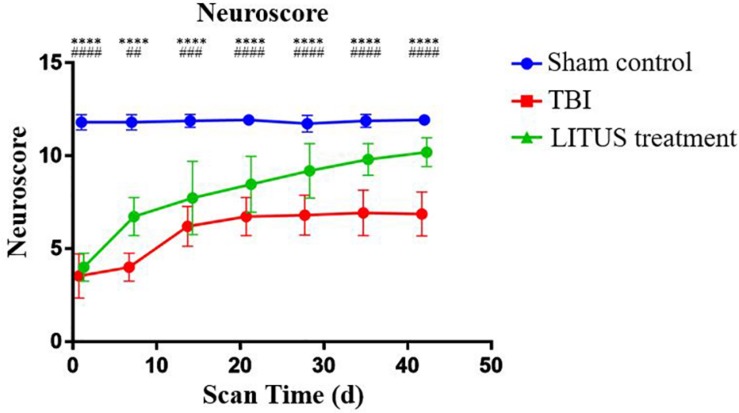
Trends of neuroscores of TBI, LITUS treatment and Sham Control groups. Two-way ANOVA for repeated measurements, followed by Tukey’s *post hoc* test. ****P < 0.0001, sham control vs. TBI. ##*P* < 0.01, ###*P* < 0.001, and ####*P* < 0.0001, TBI vs. LITUS treatment. Mean ± SD, *n* = 15.

Regarding the neuroscore in TBI, LITUS treatment, and Sham Control groups, for the control group, a baseline was maintained for 1–42 days with an average score of 11.733 to 11.933 at each time point. The scores of the TBI group and LITUS group were significantly decreased at the initial time point (day 1), 3.533 ± 1.187 and 4 ± 0.756, respectively. Within 1 to 14 days, scores improved in both the TBI group and the LITUS group, with the latter group showing significant improvement. After day 14, the TBI group’s score barely changed from 6.733 to 6.933, while the LITUS treatment group’s score improved gradually and finally reached 10.2. Statistical analysis showed that there were no statistically significant differences in neurological scores between the LITUS treatment group and the TBI group at day 1 (adjusted *P* = 0.2966), but significant differences were observed between the three groups at all other time points (all adjusted *P* < 0.0001, Figure 5).

### Immuno-Histochemical Nissl Staining

**FIGURE 6:**
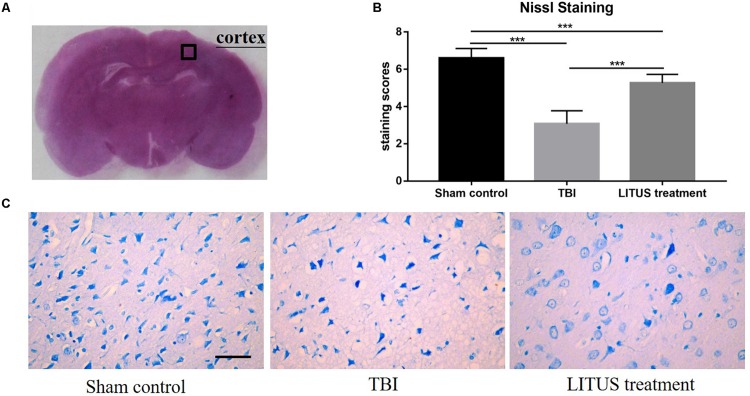
Histopathology in the ipsilateral cortex 42 days after TBI. Representative images of cresyl violet stained neurons **(A)**, Nissl staining of rat brains of TBI, LITUS treatment, and Sham Control groups at 42 days after injury. Compared to Sham Control rats, there were clear neuron edemas, the number of cells reduced, sparse arrangement, increased cell space, reduced cytoplasmic Nissl bodies, unclear boundaries, and some were dyed light blue in the TBI group. For the treatment group, nerve cells in the damaged area of rats were arranged in a dense and orderly manner, with abundant Nissl bodies in the cytoplasm, dark blue Nissl bodies in the cerebral cortex, light blue nuclei, and a light blue background **(C)**. The mean staining scores of rats in the LITUS treatment group were all between 5 and 6. The mean staining score for the Sham Control group was seven, indicating the structure of a normal nucleus in these cells **(B)**. Bar, 50 μm. Data are mean ± SD, *n* = 15. Unpaired *t*-test, ***P < 0.001.

Forty-two days after CCI in the TBI group, clear neurons edema was observed alongside a reduced number of cells, sparse arrangement, increased cell space, reduced cytoplasmic Nissl bodies, and unclear boundaries, and they were dyed light blue. Furthermore, the damaged area of the TBI rats was larger than that in the LITUS treatment group. The Nissl substance density was reduced closer to the CCI point, and the mean staining scores of the eight rats were all between two and three. However, for the treatment group, nerve cells in the damaged area of rats were arranged in a dense and orderly manner, with abundant Nissl bodies in the cytoplasm, dark blue Nissl bodies in the cerebral cortex with light blue nuclei and a light blue background. The mean staining scores of rats in the LITUS treatment group were between five and six. The mean staining score for the Sham Control group was seven, indicating a normal nucleus in these cells (Figure 6).

## Discussion

In this study, LITUS stimulation was performed in a rat model of moderate TBI. We chose this moderate TBI model instead of mild or severe TBI models because mild brain injury is milder and often accompanied with atypical clinical and pathological manifestations, which can recover by themselves after a period and thus interfere with the evaluation of LITUS treatment (Wei et al., 2012). For the severe TBI model, the degree of brain injury was severe, and the mortality of rats was quite high, and LITUS treatment is not always guaranteed to work (Le and Gean, 2010). Therefore, these two models were not selected in this study. Moderate TBI model rats often have difficulties in recovery and exhibit an appropriate degree of neurological damage, which is suitable for evaluating the treatment of LITUS (Pan et al., 2019).

Diffusion tensor imaging in brain tissue water based on molecular diffusion differences in multiple directions can quantitatively measure the reaction degree of brain edema. Various diffusion-sensitive parameters, such as FA, MD, axial diffusivity (AD), and radial diffusivity (RD), can be calculated to reflect the restricted movement of water molecules at different directions. AD and RD are only considered for axial or radial diffusion. As the analyzed areas are in the cortex, where there is no preferential diffusion direction, AD and RD were not well defined. We therefore used FA and MD there. Previous studies have also shown that traumatic brain injury can lead to significant changes in FA and MD values; therefore, these two indicators can be used to evaluate TBI (O’Phelan et al., 2018). In addition to evaluating cerebral edema, it can also reflect the integrity of white matter fiber bundles and evaluate the damage of brain tissue after trauma. We also measured the FA and MD values in the contralateral mirror region and found that there was no difference in FA and MD values between the groups and they remained in the baseline state, which confirmed the reliability of FA and MD values in evaluating the development of TBI and the effect of LITUS treatment.

In this study, we observed DTI data at multiple consecutive time points to fully reflect the pathological changes from the acute stage to a chronic stage in the rat model of TBI and avoid the one-sided conclusion that may be caused by a single time point study. We found that, in the early days after TBI (1–7 days), the FA value increased and the MD value decreased in the brain injury areas. Previous animal studies confirmed that the MD value of traumatic brain tissue continued to decrease between 60 min and 7 days after injury, and the pathological change was the main obstacle of extracellular water diffusion caused by cytotoxic edema. Similar findings have been replicated in human studies (Marmarou et al., 2006). Eierud et al. (2014) found that the FA value increased significantly and the MD value decreased significantly within 1 week after acute brain trauma, suggesting that the acute brain tissue was dominated by cytotoxic edema; additionally, since the extracellular space was narrowed, it lead to an increase in the FA value. However, the axoplasmic transport disorder in brain tissue results in a decrease in the MD value (Eierud et al., 2014). Bazarian et al. (2007) and Wilde et al. (2008) also found that, in addition to the changes in the FA and MD values in the lesion area, the differences between the FA and MD values in non-injured areas, such as the corpus callosum, can be accurately detected in some patients with acute TBI, indicating that DTI imaging can be used as a reliable method to evaluate the degree and scope of traumatic brain injury.

In this study, the FA value exhibited a decreasing trend during the recovery stage after injury, while the MD value gradually recovered. The reason may be that the FA value decreased and MD value increased with the widening of extracellular space caused by neuron apoptosis, myelin loss, and vasogenic edema. We found that the number of neurons in the TBI rats was significantly reduced by Nissl staining and that the staining was light, which confirmed the significant neuronal injury caused by TBI. O’Phelan et al. (2018) recent human study found that, while some patients with moderate TBI exhibited no abnormalities in structural MR images, the researchers found, through the quantitative DTI study, that they had extensive microstructural damage in the white matter fiber bundle area and deep nucleus group. Moreover, their FA value was significantly lower than the control group, while their MD value was significantly higher than the control group (O’Phelan et al., 2018). Previous studies have found that, for patients with moderate-to-severe TBI, pathological changes in the acute phase may persist for a long time, and progressive axonal degeneration may be detected several years after injury (Tal et al., 2017). de Lanerolle et al. (2015) confirmed this view in 43 moderate TBI patients and further found that in the chronic phase, moderate TBI patients with a poor prognosis than those with a good prognosis showed a lower FA and a higher MD value. They also proved that that DTI is a powerful method to predict the cognitive dysfunction of moderate TBI (de Lanerolle et al., 2015). In this study, the FA value in the TBI group was significantly lower than the control group at day 42, while the MD value did not show any such changes. From the perspective of animal studies, it was confirmed that the FA value might be more sensitive than MD value in evaluating long-term prognosis of TBI, suggesting that the FA value can accurately evaluate the integrity of large white matter fiber bundles (Lipton et al., 2012). Previous clinical studies have also used susceptibility weighted imaging (SWI) and other means to detect cerebral micro hemorrhage and indirectly assess the degree of axolock injury; however, none of them are more direct than DTI (Zappala et al., 2012; Toth et al., 2018).

According to a literature report, many theories have contributed to the pathogenesis of traumatic brain injury (TBI), such as blood brain barrier destruction, calcium channel dysfunction, brain microcirculation disturbance, energy metabolism abnormality, etc. None of the above theories can fully explain the pathogenesis of TBI (Zhang et al., 2018; Li et al., 2019; Svedung Wettervik et al., 2020). This is because the mechanism of TBI is the result of comprehensive action of many factors mentioned above. For example, cerebral microcirculation disturbance can aggravate ischemia and hypoxia, decrease ATP synthesis, and destroy blood–brain barrier. In addition, the increase in monoamine neurotransmitters, glutamic acid, nitric oxide, bradykinin, endothelin, and arachidonic acid also participate in the occurrence and development of traumatic brain edema (Li et al., 2015; Kozlov et al., 2017; Treble-Barna et al., 2017; Armstead and Vavilala, 2019; Morris et al., 2019). No matter what the pathogenesis of TBI are, the brain tissue edema and nerve fiber damage will occur, which will cause the FA value to decrease and the MD value to increase in DTI (Hanks et al., 2019). Therefore, if the FA value increases after an injury, it generally indicates that the injured nerve fibers have begun to be repaired, and its integrity has begun to be restored; otherwise, it indicates that the brain tissue edema and the nerve fiber damage is being aggravated. In similar mechanisms, a decrease of MD value also indicates the decrease of edema, which generally means an alleviation of TBI; otherwise, it generally indicates that the edema is increasing and the TBI is being aggravated.

LITUS, as a rapidly developing encephalopathy physiotherapy in the last 10 years, has unique advantages in the treatment of epilepsy, brain degenerative diseases, and mental diseases (Hameroff et al., 2013; Panczykowski et al., 2014; Tsai, 2015). It can affect the living environment of neurons by regulating molecular signal transduction, enhancing the expression of nutrients, and regulating the release of neuro-electrical signals. Previous researchers have mostly explored the regulatory effects of LITUS at the pathological and molecular levels. Our previous studies have found that LITUS can carry on the regulation of the brain microenvironment, and, by further application LITUS model of middle cerebral artery embolization treatment in rats, we found that early application LITUS could alleviate the occurrence of cytotoxic brain edema after acute cerebral infarction, and its mechanism may be through the expression of BDNF. This study further explored the therapeutic effect of LITUS on TBI from a neuroimaging perspective. In our study, we found that FA values increased and MD values decreased in the early stage (1 day after injury) of the LITUS treated group. With the increase of treatment times and the accumulation of treatment effects, FA and MD values gradually reached a similar level without significant difference compared to contrlateral healthy side at the end of the study. The changing trend of FA and MD values was consistent with the therapeutic effect of LITUS, where LITUS has been confirmed in previous studies to inhibit neuronal edema, apoptosis, and gliosis by enhancing the expression of neurotrophic factors and other substances through neuron staining method (Su et al., 2015, 2017a). Through our study, we found the DTI-derived parameters FA and MD may play a role as potential bio-markers in evaluation of the therapeutic effect of LITUS. In addition, we found that compared to TBI rats, the nerve score of the treatment group was significantly better than that of the injury group. Meanwhile, we used Nissl staining to confirm that, in contrast to the obvious damage of TBI rats’ neurons, LITUS treatment can alleviate the degeneration of neurons and play a neuroprotective role. These two results combined suggested that continued LITUS therapy improves the behavioral and histopathological outcomes of rats with TBI.

Previous animal studies have shown that the subcellular changes in local nerve tissue, caused by brain trauma, are often reversible in causing focal neurological deficits (Pasco et al., 2007). This serves as a structural basis for LITUS regulation. As for the mechanism of LITUS regulation on TBI, research conclusions are not consistent. Some researchers believe that LITUS can promote the opening of the blood–brain barrier and thus reduce the infiltration of inflammatory cells (Su et al., 2017b). They found that early application of LITUS stimulation could significantly reduce the expression of brain injury-related proteins in TBI mice, improve BBB permeability, and prevent the common sequential inflammatory response in the brain after TBI, thereby also improving the long-term prognosis of TBI mice (Su et al., 2017b).

Cellular and molecular changes can be caused by LITUS. LITUS may promote the rehabilitation of TBI at both cellular and molecular levels. At the cellular level, researchers believe that LITUS can regulate cell function at the ultrastructural level. When TBI occurs, the axon membrane calcium ion channel under the action of mechanical force is triggered, which leads to calcium influx caused by a variety of dependent on the enzymatic reaction of Ca2 +, functional and structural damage to the axonal cytoskeleton, altered axoplasmic transport, and swelling in the axon. LITUS can work on drawing a form of calcium channel, which leads to increased divalent calcium and thereby decreasing calcium current. This can improve the function of the calcium pump, inhibition of axonal injury, and lead to decreased FA values. In addition, LITUS can also regulate the dysfunction of Na + -k + pump on the axial membrane, inhibit cytotoxic edema, and increase MD values (Buki et al., 1999). At the molecular level, some researchers have pointed out that LITUS is modulated by the regulation of the transduction of molecular signals and the enhancement of the expression of neuronutrients like BDNF. By monitoring ion conductance and exocytosis of prominent vesicles, Reher et al. (1999) found that LITUS could affect nerve function by modulating the generation and transmission of cell signaling molecules. Subsequent studies have found that LITUS can stimulate the production of various cytokines, such as bFGF, TGF-b, BMP-7, VEGF, and igf-1 (Reher et al., 1999; Naruse et al., 2003; Mukai et al., 2005; Sant’Anna et al., 2005), thus specifically affecting nerve growth and survival, axonal guidance, and angiogenesis. In addition to promoting the transfer of molecular signals, LITUS can also promote the expression of BDNF. Chen et al. (2018) found that LITUS pre-stimulation could increase the expression of BDNF, thus preventing degenerative damage to neurons. It is well known that the increased expression of BDNF can promote the recovery of nerve function, reduce the production of oxygen free radicals, and prevent cerebral edema (Chen et al., 2018).

However, up to now, researchers still argue over the regulatory mechanism of PD. FA and MD values adopted in this study reflect the integrity of nerve axons and the state of cell edema to a certain extent. Therefore, we believe that LITUS could play an important role in promoting anti-axonal injury and inhibiting brain edema. Given the multiplicity of LITUS’ neuro-regulatory mechanisms, LITUS is likely to regulate the rehabilitation process of brain trauma from multiple dimensions. This study, mainly from the perspective of neuroimaging, has verified the significant effect of continuous LITUS application on promoting neurological rehabilitation and has provided evidence for its possible mechanism.

There are a few limitations to this study. First of all, although special rat coils were used in this study for image collection, the MR equipment was a 3T MR machine instead of a magnetic resonance machine for small animals, and this lead to unsatisfactory imaging quality, which was one of the main limitations of this study. Second, all the ROIs were detected manually, and, therefore, the location and size of the ROIs may have differed between rats or groups in the present single-layer measurement. Third, due to the limitation of the experimental condition, the brain injury area could not be divided into central and surrounding areas and thus measured separately, which may have compromised the quality of our results. However, with advances in MR and image processing techniques, DTI may play a more important role in monitoring and evaluating the efficacy of drug therapy in the future. We also found that, although LITUS could promote the recovery of neurological function scores in rats, we only used a single fixation of the therapeutic parameter. Therefore, in the studies study, one should try to optimize the parameters of ultrasound stimulation or combine them with other drug treatments in order to further improve the efficacy of ultrasound stimulation.

## Conclusion

In conclusion, this study demonstrated that treatment with LITUS daily for 10 min effectively improve the brain damage via multiple methods as DTI parameters, a neuroscore, and Nissl’s Staining in a CCI-caused TBI model. LITUS treatment can prevent nerve fiber apoptosis, maintain the integrity of the myelin sheath, and improve the functional prognosis of TBI. These findings suggested that LITUS treatment may be potentially used to prevent TBI or even another neuronal disease and serve as a novel strategy in a clinical setting in the future.

## Data Availability Statement

The raw data supporting the conclusions of this article will be made available by the authors, without undue reservation, to any qualified researcher.

## Ethics Statement

The animal study was reviewed and approved by the Medical Ethics Committee of Qinhuangdao Municipal No. 1 Hospital.

## Author Contributions

TZ, YY, YJ, and LL designed and coordinated the study. TZ, JD, SW, ZW, DL, XW, and QS carried out the experiment and data processing and drafted the manuscript. All authors gave final approval for publication.

## Conflict of Interest

QS was employed by the company Siemens Ltd. The remaining authors declare that the research was conducted in the absence of any commercial or financial relationships that could be construed as a potential conflict of interest.

## References

[B1] Armstead W. M. Vavilala M. S. (2019). Translational approach towards determining the role of cerebral autoregulation in outcome after traumatic brain injury. *Exp. Neurol.* 317 291–297. 10.1016/j.expneurol.2019.03.015 30928388 PMC6544502

[B2] Axiotis C. A. Monteagudo C. Merino M. J. LaPorte N. Neumann R. D. (1991). Immunohistochemical detection of P-glycoprotein in endometrial adenocarcinoma. *Am. J. Pathol.* 138 799–806. 1707232 PMC1886115

[B3] Bazarian J. J. Zhong J. Blyth B. Zhu T. Kavcic V. Peterson D. (2007). Diffusion tensor imaging detects clinically important axonal damage after mild traumatic brain injury: a pilot study. *J. Neurotrauma* 24 1447–1459. 10.1089/neu.2007.0241 17892407

[B4] Buki A. Siman R. Trojanowski J. Q. Povlishock J. T. (1999). The role of calpain-mediated spectrin proteolysis in traumatically induced axonal injury. *J. Neuropathol. Exp. Neurol.* 58 365–375. 10.1097/00005072-199904000-00007 10218632

[B5] Cascio C. J. Gerig G. Piven J. (2007). Diffusion tensor imaging: application to the study of the developing brain. *J. Am. Acad. Child Adolesc. Psychiatry* 46 213–223. 17242625 10.1097/01.chi.0000246064.93200.e8

[B6] Chen C. M. Wu C. T. Yang T. H. Liu S. H. Yang F. Y. (2018). Preventive effect of low intensity pulsed ultrasound against experimental cerebral ischemia/reperfusion injury via apoptosis reduction and brain-derived neurotrophic factor induction. *Sci. Rep.* 8:5568. 10.1038/s41598-018-23929-8 29615782 PMC5882812

[B7] de Lanerolle N. C. Kim J. H. Bandak F. A. (2015). Neuropathology of traumatic brain injury: comparison of penetrating, nonpenetrating direct impact and explosive blast etiologies. *Semin. Neurol.* 35 12–19. 10.1055/s-0035-1544240 25714863

[B8] Eierud C. Craddock R. C. Fletcher S. Aulakh M. King-Casas B. Kuehl D. (2014). Neuroimaging after mild traumatic brain injury: review and meta-analysis. *Neuroimage Clin.* 4 283–294. 10.1016/j.nicl.2013.12.009 25061565 PMC4107372

[B9] Hameroff S. Trakas M. Duffield C. Annabi E. Gerace M. B. Boyle P. (2013). Transcranial ultrasound (TUS) effects on mental states: a pilot study. *Brain Stimul.* 6 409–415. 10.1016/j.brs.2012.05.002 22664271

[B10] Hanks R. Millis S. Scott S. Gattu R. O’Hara N. B. Haacke M. (2019). The relation between cognitive dysfunction and diffusion tensor imaging parameters in traumatic brain injury. *Brain Inj.* 33 355–363. 10.1080/02699052.2018.1553073 30563361

[B11] Hayes J. P. Logue M. W. Sadeh N. Spielberg J. M. Verfaellie M. Hayes S. M. (2017). Mild traumatic brain injury is associated with reduced cortical thickness in those at risk for Alzheimer”s disease. *Brain* 140:813. 10.1093/brain/aww344 28077398 PMC6075586

[B12] Immonen R. J. Kharatishvili I. Niskanen J. P. Grohn H. Pitkanen A. Grohn O. H. (2009). Distinct MRI pattern in lesional and perilesional area after traumatic brain injury in rat–11 months follow-up. *Exp. Neurol.* 215 29–40. 10.1016/j.expneurol.2008.09.009 18929562

[B13] Joseph B. Pandit V. Aziz H. Kulvatunyou N. Zangbar B. Green D. J. (2015). Mild traumatic brain injury defined by glasgow coma scale: is it really mild? *Brain Inj.* 29 11–16. 10.3109/02699052.2014.945959 25111571

[B14] Kowalski R. G. Haarbauer-Krupa J. K. Bell J. M. Corrigan J. D. Hammond F. M. Torbey M. T. (2017). Acute ischemic stroke after moderate to severe traumatic brain injury: incidence and impact on outcome. *Stroke* 48 1802–1809. 10.1161/STROKEAHA.117.017327 28611087 PMC6025795

[B15] Kozlov A. V. Bahrami S. Redl H. Szabo C. (2017). Alterations in nitric oxide homeostasis during traumatic brain injury. *Biochim. Biophys. Acta Mol. Basis Dis.* 1863 2627–2632. 10.1016/j.bbadis.2016.12.020 28064018

[B16] Le T. H. Gean A. D. (2010). Neuroimaging of traumatic brain injury. *Mount Sin. J. Medi. J. Trans. Personal. Medi.* 76 145–162. 10.1002/msj.20102 19306377

[B17] Li B. Tian J. P. Zhang S. Guo Y. Tu Y. Yi T. L. (2019). Effect of bloodletting acupuncture at twelve jing-well points of hand on microcirculatory disturbance in mice with traumatic brain injury. *Zhongguo Zhen Jiu* 39 1075–1080. 10.13703/j.0255-2930.2019.10.012 31621260

[B18] Li W. T. Zhang S. Y. Zhou Y. F. Zhang B. F. Liang Z. Q. Liu Y. H. (2015). Carvacrol attenuates traumatic neuronal injury through store-operated Ca(2+) entry-independent regulation of intracellular Ca(2+) homeostasis. *Neurochem. Int.* 90 107–113. 10.1016/j.neuint.2015.07.020 26220904

[B19] Lipton M. L. Kim N. Park Y. K. Hulkower M. B. Gardin T. M. Shifteh K. (2012). Robust detection of traumatic axonal injury in individual mild traumatic brain injury patients: intersubject variation, change over time and bidirectional changes in anisotropy. *Brain Imaging Behav.* 6 329–342. 10.1007/s11682-012-9175-2 22684769

[B20] Liu L. Du J. Zheng T. Hu S. Zhao M. Wang X. (2019). Readout-segmented echo-planar diffusion-weighted MR at 3.0T for the evaluation the effect of low-intensity transcranial ultrasound on stroke in a rat model. *Magn. Reson. Imaging* 67 79–84. 10.1016/j.mri.2019.06.013 31278999

[B21] Marmarou A. Signoretti S. Fatouros P. P. Portella G. Aygok G. A. Bullock M. R. (2006). Predominance of cellular edema in traumatic brain swelling in patients with severe head injuries. *J. Neurosurg.* 104 720–730. 10.3171/jns.2006.104.5.720 16703876

[B22] Monti M. M. Schnakers C. Korb A. S. Bystritsky A. Vespa P. M. (2016). Non-invasive ultrasonic thalamic stimulation in disorders of consciousness after severe brain injury: a first-in-man report. *Brain Stimul.* 9 940–941. 10.1016/j.brs.2016.07.008 27567470

[B23] Morris M. C. Kassam F. Bercz A. Beckmann N. Schumacher F. Gulbins E. (2019). The role of chemoprophylactic agents in modulating platelet aggregability after traumatic brain injury. *J. Surg. Res.* 244 1–8. 10.1016/j.jss.2019.06.022 31279258 PMC6815704

[B24] Mukai S. Ito H. Nakagawa Y. Akiyama H. Miyamoto M. Nakamura T. (2005). Transforming growth factor-beta1 mediates the effects of low-intensity pulsed ultrasound in chondrocytes. *Ultrasound. Med. Biol.* 31 1713–1721. 10.1016/j.ultrasmedbio.2005.07.012 16344134

[B25] Naruse K. Miyauchi A. Itoman M. Mikuni-Takagaki Y. (2003). Distinct anabolic response of osteoblast to low-intensity pulsed ultrasound. *J. Bone Miner. Res.* 18 360–369. 10.1359/jbmr.2003.18.2.360 12568414

[B26] O’Phelan K. H. Otoshi C. K. Ernst T. Chang L. (2018). Common patterns of regional brain injury detectable by diffusion tensor imaging in otherwise normal-appearing white matter in patients with early moderate to severe traumatic brain injury. *J. Neurotrauma.* 35 739–749. 10.1089/neu.2016.4944 29228858 PMC5831746

[B27] Pan M. X. Tang J. C. Liu R. Feng Y. G. Wan Q. (2019). Effects of estrogen receptor GPR30 agonist G1 on neuronal apoptosis and microglia polarization in traumatic brain injury rats. *Chin. J. Traumatol.* 21 224–228. 10.1016/j.cjtee.2018.04.003 30017543 PMC6085194

[B28] Panczykowski D. M. Monaco E. A. III Friedlander R. M. (2014). Transcranial focused ultrasound modulates the activity of primary somatosensory cortex in humans. *Neurosurgery* 74:N8. 10.1227/NEU.0000000000000365 24836106

[B29] Pasco A. Lemaire L. Franconi F. Lefur Y. Noury F. Saint-Andre J. P. (2007). Perfusional deficit and the dynamics of cerebral edemas in experimental traumatic brain injury using perfusion and diffusion-weighted magnetic resonance imaging. *J Neurotrauma* 24 1321–1330. 10.1089/neu.2006.0136 17711393 PMC3218539

[B30] Reher P. Doan N. Bradnock B. Meghji S. Harris M. (1999). Effect of ultrasound on the production of IL-8, basic FGF and VEGF. *Cytokine* 11 416–423. 10.1006/cyto.1998.0444 10346981

[B31] Sant’Anna E. F. Leven R. M. Virdi A. S. Sumner D. R. (2005). Effect of low intensity pulsed ultrasound and BMP-2 on rat bone marrow stromal cell gene expression. *J. Orthop. Res.* 23 646–652. 10.1016/j.orthres.2004.09.007 15885487

[B32] Scott L. K. Green R. Mccarthy P. J. Conrad S. A. (2009). Agitation and/or aggression after traumatic brain injury in the pediatric population treated with ziprasidone. Clinical article. *J. Neurosurg. Pediatr.* 3:484. 10.3171/2009.2.PEDS08292 19485732

[B33] Stuart H. Michael T. Chris D. Emil A. Bagambhrini G. M. Patrick B. (2013). Transcranial ultrasound (TUS) effects on mental states: a pilot study. *Brain Stimul.* 6 409–415. 22664271 10.1016/j.brs.2012.05.002

[B34] Su W. S. Tsai M. L. Huang S. L. Liu S. H. Yang F. Y. (2015). Controllable permeability of blood-brain barrier and reduced brain injury through low-intensity pulsed ultrasound stimulation. *Oncotarget* 6 42290–42299. 10.18632/oncotarget.5978 26517350 PMC4747225

[B35] Su W. S. Wu C. H. Chen S. F. Yang F. Y. (2017a). Low-intensity pulsed ultrasound improves behavioral and histological outcomes after experimental traumatic brain injury. *Sci. Rep.* 7:15524. 10.1038/s41598-017-15916-2 29138458 PMC5686128

[B36] Su W. S. Wu C. H. Chen S. F. Yang F. Y. (2017b). Transcranial ultrasound stimulation promotes brain-derived neurotrophic factor and reduces apoptosis in a mouse model of traumatic brain injury. *Brain Stimul.* 10 1032–1041. 10.1016/j.brs.2017.09.003 28939348

[B37] Svedung Wettervik T. Howells T. Hillered L. Nilsson P. Engquist H. Lewen A. (2020). Mild hyperventilation in traumatic brain injury-relation to cerebral energy metabolism. Pressure autoregulation, and clinical outcome. *World Neurosurg.* 133 e567–e575. 10.1016/j.wneu.2019.09.099 31561041

[B38] Tal S. Hadanny A. Sasson E. Suzin G. Efrati S. (2017). Hyperbaric oxygen therapy can induce angiogenesis and regeneration of nerve fibers in traumatic brain injury patients. *Front. Hum. Neurosci.* 11:508. 10.3389/fnhum.2017.00508 29097988 PMC5654341

[B39] Toth A. Kornyei B. Kovacs N. Rostas T. Buki A. Doczi T. (2018). Both hemorrhagic and non-hemorrhagic traumatic MRI lesions are associated with the microstructural damage of the normal appearing white matter. *Behav. Brain Res.* 340 106–116. 10.1016/j.bbr.2017.02.039 28249729

[B40] Treble-Barna A. Wade S. L. Martin L. J. Pilipenko V. Yeates K. O. Taylor H. G. (2017). Influence of dopamine-related genes on neurobehavioral recovery after traumatic brain injury during early childhood. *J. Neurotrauma* 34 1919–1931. 10.1089/neu.2016.4840 28323555 PMC5455258

[B41] Tsai S. J. (2015). Transcranial focused ultrasound as a possible treatment for major depression. *Med. Hypotheses* 84 381–383. 10.1016/j.mehy.2015.01.030 25665863

[B42] Wei X. E. Zhang Y. Z. Li Y. H. Li M. H. Li W. B. (2012). Dynamics of rabbit brain edema in focal lesion and perilesion area after traumatic brain injury: a MRI study. *J. Neurotrauma* 29 2413–2420. 10.1089/neu.2010.1510 21675826 PMC3433692

[B43] Wilde E. A. McCauley S. R. Hunter J. V. Bigler E. D. Chu Z. Wang Z. J. (2008). Diffusion tensor imaging of acute mild traumatic brain injury in adolescents. *Neurology* 70 948–955. 10.1212/01.wnl.0000305961.68029.54 18347317

[B44] Williams W. H. Chitsabesan P. Fazel S. McMillan T. Hughes N. Parsonage M. (2018). Traumatic brain injury: a potential cause of violent crime? *Lancet. Psychiatry* 5 836–844. 10.1016/s2215-0366(18)30062-2 29496587 PMC6171742

[B45] Xiong K. Zhu Y. Zhang Y. Yin Z. Zhang J. Qiu M. (2014). White matter integrity and cognition in mild traumatic brain injury following motor vehicle accident. *Brain Res.* 1591 86–92. 10.1016/j.brainres.2014.10.030 25451093

[B46] Yuan Y. Dong Y. Hu S. Zheng T. Du D. Du J. (2017). Reduced apparent diffusion coefficient in various brain areas following low-intensity transcranial ultrasound stimulation. *Front. Neurosci.* 11:562. 10.3389/fnins.2017.00562 29062269 PMC5640877

[B47] Zappala G. Thiebaut de Schotten M. Eslinger P. J. (2012). Traumatic brain injury and the frontal lobes: what can we gain with diffusion tensor imaging? *Cortex* 48 156–165. 10.1016/j.cortex.2011.06.020 21813118

[B48] Zetterberg H. Smith D. H. Blennow K. (2013). Biomarkers of mild traumatic brain injury in cerebrospinal fluid and blood. *Nat. Rev. Neurol.* 9 201–210. 10.1038/nrneurol.2013.9 23399646 PMC4513656

[B49] Zhang M. Wu Y. Xie L. Teng C. H. Wu F. F. Xu K. B. (2018). Corrigendum to “Isoliquiritigenin protects against blood-brain barrier damage and inhibits the secretion of pro-inflammatory cytokines in mice after traumatic brain injury” [International Immunopharmacology 65 64-75]. *Int. Immunopharmacol.* 67:490. 10.1016/j.intimp.2018.12.038 30598397

