# Supplementary material for: Neuroprotective Effect of Low-Intensity Transcranial Ultrasound Stimulation in Moderate Traumatic Brain Injury Rats
Source: Front Neurosci. 2020 Mar 10;14:172. doi: 10.3389/fnins.2020.00172 (PMC7078644; doi:10.3389/fnins.2020.00172)
Supplement: Supplementary file 1 [file Data_Sheet_1.docx]

**Supplement Materials**

| Supplement table 1. FA values at the focal lesion of the Sham Control (SC), TBI and LITUS treatment groups | | | | | | |
| --- | --- | --- | --- | --- | --- | --- |
|  | SC VS. TBI（n=15） | Adjusted P | SC VS. LITUS（n=15） | Adjusted P | TBI VS. LITUS（n=15） | Adjusted P |
| FA at day 1 | 0.3445±0.132 VS. 0.491±0.172 | 0.0202* | 0.3445±0.132VS. 0.475±0.121 | 0.0422* | 0.491±0.172 VS. 0.475±0.121 | 0.9499 |
|  |  |  |  |  |  |  |
| FA at day 7 | 0.341±0.145 VS. 0.522±0.115 | 0.0017** | 0.341±0.145VS. 0.436±0.138 | 0.1369 | 0.522±0.115 VS. 0.436±0.138 | 0.193 |
|  |  |  |  |  |  |  |
| FA at day 14 | 0.345±0.127 VS. 0.512±0.129 | 0.0009*** | 0.345±0.127 VS. 0.352±0.090 | 0.9852 | 0.512±0.129 VS. 0.352±0.090 | 0.0015** |
|  |  |  |  |  |  |  |
| FA at day 21 | 0.288±0.096 VS. 0.483±0.141 | 0.0001*** | 0.288±0.096 VS. 0.346±0.105 | 0.3637 | 0.483±0.141 VS. 0.346±0.105 | 0.0064** |
|  |  |  |  |  |  |  |
| FA at day 28 | 0.294±0.139 VS. 0.454±0.094 | 0.0006*** | 0.294±0.139 VS. 0.375±0.084 | 0.1135 | 0.454±0.094 VS. 0.375±0.084 | 0.1255 |
|  |  |  |  |  |  |  |
| FA at day 35 | 0.301±0.101 VS. 0.378±0.171 | 0.271 | 0.301±0.101 VS. 0.237±0.122 | 0.4018 | 0.378±0.171 VS. 0.237±0.122 | 0.0173* |
|  |  |  |  |  |  |  |
| FA at day 42 | 0.361±0.096 VS. 0.228±0.068 | 0.0014** | 0.361±0.096 VS. 0.31±0.12 | 0.3242 | 0.228±0.068 VS. 0.31±0.12 | 0.0619 |
|  |  |  |  |  |  |  |
| Values are represented as mean±SD. | | | | | | |

| Supplement table 2. MD Values(× 10–3 mm2/s) at the focal lesion of the Sham Control (SC), TBI and LITUS treatment groups | | | | | | |
| --- | --- | --- | --- | --- | --- | --- |
|  | SC VS. TBI（n=15） | Adjusted P | SC VS. LITUS（n=15） | Adjusted P | TBI VS. LITUS（n=15） | Adjusted P |
| MD at day 1 | 0.842±0.238 VS. 0.463±0.310 | 0.0007*** | 0.842±0.238 VS. 0.735±0.218 | 0.4988 | 0.463±0.310 VS. 0.735±0.218 | 0.0167* |
|  |  |  |  |  |  |  |
| MD at day 7 | 0.816±0.261 VS. 0.878±0.207 | 0.76 | 0.816±0.261 VS. 0.982±0.248 | 0.1524 | 0.878±0.207 VS. 0.982±0.248 | 0.4669 |
|  |  |  |  |  |  |  |
| MD at day 14 | 0.801±0.229 VS. 1.037±0.232 | 0.0101* | 0.801±0.229 VS. 0.872±0.162 | 0.6276 | 1.037±0.232 VS. 0.872±0.162 | 0.092 |
|  |  |  |  |  |  |  |
| MD at day 21 | 0.789±0.173 VS. 1.098±0.254 | 0.0006*** | 0.789±0.173 VS. 0.811±0.189 | 0.955 | 1.098±0.254 VS. 0.811±0.189 | 0.0014** |
|  |  |  |  |  |  |  |
| MD at day 28 | 0.809±0.250 VS. 1.159±0.169 | <0.0001*** | 0.809±0.2501 VS. 0.923±0.15 | 0.2558 | 1.159±0.169 VS. 0.923±0.151 | 0.0052* |
|  |  |  |  |  |  |  |
| MD at day 35 | 0.800±0.182 VS. 1.180±0.308 | 0.0003*** | 0.800±0.182 VS. 0.952±0.220 | 0.2109 | 1.180±0.308 VS. 0.952±0.220 | 0.0356* |
|  |  |  |  |  |  |  |
| MD at day 42 | 0.806±0.173 VS. 1.215±0.122 | <0.0001*** | 0.806±0.173 VS. 0.872±0.212 | 0.5531 | 1.215±0.122 VS. 0.872±0.212 | <0.0001*** |
|  |  |  |  |  |  |  |
| Values are represented as mean±SD. | | | | | | |

| Supplement table 3. FA values at the contralateral mirror cortex of the Sham Control (SC), TBI and LITUS treatment groups | | | | | | |
| --- | --- | --- | --- | --- | --- | --- |
|  | SC VS. TBI（n=15） | Adjusted P | SC VS. LITUS（n=15） | Adjusted P | TBI VS. LITUS（n=15） | Adjusted P |
| FA at day 1 | 0.358±0.158 VS. 0.290±0.202 | 0.4158 | 0.358±0.158 VS. 0.335±0.146 | 0.9040 | 0.290±0.202 VS. 0.335±0.146 | 0.6800 |
|  |  |  |  |  |  |  |
| FA at day 7 | 0.325±0.173 VS. 0.288±0.140 | 0.7703 | 0.325±0.173 VS. 0.407±0.165 | 0.2801 | 0.288±0.140 VS. 0.407±0.165 | 0.0703 |
|  |  |  |  |  |  |  |
| FA at day 14 | 0.329±0.153 VS. 0.279±0.155 | 0.6213 | 0.329±0.153 VS. 0.404±0.112 | 0.3443 | 0.279±0.155 VS. 0.404±0.112 | 0.0537 |
|  |  |  |  |  |  |  |
| FA at day 21 | 0.277±0.119 VS. 0.283±0.168 | 0.9931 | 0.277±0.119 VS. 0.388±0.129 | 0.0998 | 0.283±0.168 VS. 0.388±0.129 | 0.1255 |
|  |  |  |  |  |  |  |
| FA at day 28 | 0.283±0.166 VS. 0.327±0.116 | 0.6916 | 0.283±0.166 VS. 0.380±0.105 | 0.1696 | 0.327±0.116 VS. 0.380±0.105 | 0.5860 |
|  |  |  |  |  |  |  |
| FA at day 35 | 0.289±0.124 VS. 0.328±0.201 | 0.7483 | 0.289±0.124 VS. 0.328±0.147 | 0.7483 | 0.328±0.201 VS. 0.328±0.147 | ＞0.9999 |
|  |  |  |  |  |  |  |
| FA at day 42 | 0.343±0.119 VS. 0.301±0.088 | 0.7145 | 0.343±0.119 VS. 0.338±0.143 | 0.9952 | 0.301±0.088 VS. 0.338±0.143 | 0.7703 |
|  |  |  |  |  |  |  |
| Values are represented as mean±SD. | | | | | | |

| Supplement table 4. MD Values(× 10–3 mm2/s) at the contralateral mirror cortex of the Sham Control (SC), TBI and LITUS treatment groups | | | | | | |
| --- | --- | --- | --- | --- | --- | --- |
|  | SC VS. TBI（n=15） | Adjusted P | SC VS. LITUS（n=15） | Adjusted P | TBI VS. LITUS（n=15） | Adjusted P |
| MD at day 1 | 0.974±0.301 VS. 0.870±0.447 | 0.5510 | 0.974±0.238 VS. 0.905±0.262 | 0.7454 | 0.870±0.447 VS. 0.905±0.262 | 0.9271 |
|  |  |  |  |  |  |  |
| MD at day 7 | 0.975±0.351 VS. 0.864±0.242 | 0.5074 | 0.975±0.261 VS. 1.021±0.325 | 0.8774 | 0.864±0.242 VS. 1.021±0.325 | 0.2213 |
|  |  |  |  |  |  |  |
| MD at day 14 | 0.967±0.285 VS. 0.757±0.292 | 0.0908 | 0.967±0.229 VS. 0.912±0.150 | 0.8296 | 0.757±0.292 VS. 0.912±0.150 | 0.2298 |
|  |  |  |  |  |  |  |
| MD at day 21 | 0.831±0.173 VS. 0.819±0.334 | 0.9921 | 0.831±0.173 VS. 0.964±0.206 | 0.3377 | 0.819±0.334 VS. 0.964±0.206 | 0.2756 |
|  |  |  |  |  |  |  |
| MD at day 28 | 0.849±0.328 VS. 0.881±0.163 | 0.9449 | 0.849±0.250 VS. 0.914±0.127 | 0.7704 | 0.881±0.163 VS. 0.914±0.127 | 0.9349 |
|  |  |  |  |  |  |  |
| MD at day 35 | 0.887±0.189 VS. 0.884±0.443 | 0.9995 | 0.887±0.182 VS. 0.984±0.265 | 0.5601 | 0.884±0.443 VS. 0.984±0.265 | 0.5402 |
|  |  |  |  |  |  |  |
| MD at day 42 | 0.929±0.173 VS. 0.903±0.070 | 0.9633 | 0.929±0.173 VS. 1.014±0.252 | 0.6405 | 0.903±0.070 VS. 1.014±0.252 | 0.4686 |
|  |  |  |  |  |  |  |
| Values are represented as mean±SD. | | | | | | |

| Supplement table 5. FA values at the focal lesion and contralateral mirror cortex of the Sham Control (SC), TBI and LITUS treatment groups | | | | | | |
| --- | --- | --- | --- | --- | --- | --- |
|  | SC_f_ VS. SC_c_（n=15） | Adjusted P | TBI_f_ VS. TBI_c_（n=15） | Adjusted P | LITUS_f_ VS. LITUS_c_（n=15） | Adjusted P |
| FA at day 1 | 0.344±0.132 VS. 0.358±0.158 | >0.9999 | 0.491±0.172 VS. 0.290±0.202 | 0.0014^**^ | 0.475±0.121 VS. 0.335±0.146 | 0.0174^*^ |
|  |  |  |  |  |  |  |
| FA at day 7 | 0.341±0.145 VS. 0.325±0.173 | >0.9999 | 0.522±0.115 VS. 0.288±0.140 | 0.0001^***^ | 0.436±0.138 VS. 0.407±0.165 | 0.9947 |
|  |  |  |  |  |  |  |
| FA at day 14 | 0.345±0.127 VS. 0.329±0.153 | >0.9999 | 0.512±0.129 VS. 0.279±0.155 | 0.0001^***^ | 0.352±0.090 VS. 0.404±0.112 | 0.8747 |
|  |  |  |  |  |  |  |
| FA at day 21 | 0.288±0.096 VS. 0.277±0.119 | >0.9999 | 0.483±0.141 VS. 0.283±0.168 | 0.0015^**^ | 0.346±0.105 VS. 0.388±0.129 | 0.9557 |
|  |  |  |  |  |  |  |
| FA at day 28 | 0.294±0.139 VS. 0.283±0.166 | >0.9999 | 0.454±0.094 VS. 0.327±0.116 | 0.1179 | 0.375±0.084 VS. 0.380±0.105 | >0.9999 |
|  |  |  |  |  |  |  |
| FA at day 35 | 0.301±0.101 VS. 0.289±0.124 | >0.9999 | 0.378±0.171 VS. 0.328±0.201 | 0.9498 | 0.237±0.122 VS. 0.328±0.147 | 0.2910 |
|  |  |  |  |  |  |  |
| FA at day 42 | 0.361±0.096 VS. 0.343±0.119 | 0.9998 | 0.288±0.068 VS. 0.301±0.088 | 0.7308 | 0.310±0.118 VS. 0.338±0.143 | 0.9957 |
|  |  |  |  |  |  |  |
| Values are represented as mean±SD. | | | | | | |

| Supplement table 6. MD values at the focal lesion and contralateral mirror cortex of the Sham Control (SC), TBI and LITUS treatment groups | | | | | | |
| --- | --- | --- | --- | --- | --- | --- |
|  | SC_f_ VS. SC_c_（n=15） | Adjusted P | TBI_f_ VS. TBI_c_（n=15） | Adjusted P | LITUS_f_ VS. LITUS_c_（n=15） | Adjusted P |
| MD at day 1 | 0.842±0.301 VS. 0.974±0.238 | 0.6504 | 0.463±0.310 VS. 0.870±0.447 | 0.0006^***^ | 0.735±0.218 VS. 0.905±0.262 | 0.2224 |
|  |  |  |  |  |  |  |
| MD at day 7 | 0.816±0.351 VS. 0.975±0.261 | 0.4223 | 0.878±0.207 VS. 0.864±0.242 | >0.9999 | 0.982±0.248 VS. 1.021±0.325 | 0.9990 |
|  |  |  |  |  |  |  |
| MD at day 14 | 0.801±0.285 VS. 0.967±0.229 | 0.3682 | 1.037±0.232 VS. 0.757±0.292 | 0.0431^*^ | 0.872±0.162 VS. 0.912±0.150 | 0.9988 |
|  |  |  |  |  |  |  |
| MD at day 21 | 0.789±0.173 VS. 0.831±0.173 | 0.9992 | 1.098±0.254 VS. 0.819±0.334 | 0.0443 | 0.811±0.189 VS. 0.964±0.206 | 0.3413 |
|  |  |  |  |  |  |  |
| MD at day 28 | 0.809±0.328 VS. 0.849±0.250 | 0.9994 | 1.159±0.169 VS. 0.881±0.163 | 0.0456^*^ | 0.923±0.151 VS. 0.914±0.127 | >0.9999 |
|  |  |  |  |  |  |  |
| MD at day 35 | 0.800±0.189 VS. 0.887±0.182 | 0.9389 | 1.180±0.308 VS. 0.884±0.443 | 0.0270^*^ | 0.952±0.220 VS. 0.984±0.265 | 0.9997 |
|  |  |  |  |  |  |  |
| MD at day 42 | 0.806±0.173 VS. 0.929±0.173 | 0.7247 | 1.215±0.122 VS. 0.903±0.070 | 0.0165^*^ | 0.872±0.212 VS. 1.014±0.252 | 0.4344 |
|  |  |  |  |  |  |  |
| Values are represented as mean±SD. | | | | | | |
